# Emerging SiC Applications beyond Power Electronic Devices

**DOI:** 10.3390/mi14061200

**Published:** 2023-06-06

**Authors:** Francesco La Via, Daniel Alquier, Filippo Giannazzo, Tsunenobu Kimoto, Philip Neudeck, Haiyan Ou, Alberto Roncaglia, Stephen E. Saddow, Salvatore Tudisco

**Affiliations:** 1 CNR-IMM, Strada VIII, 5, 95121 Catania, Italy; filippo.giannazzo@imm.cnr.it; 2 GREMAN, UMR 7347, Université de Tours, CNRS, 37071 Tours, France; daniel.alquier@univ-tours.fr; 3 Department of Electronic Science and Engineering, Kyoto University, Nishikyo, Kyoto 615-8510, Japan; kimoto@kuee.kyoto-u.ac.jp; 4 NASA Glenn Research Center, 21000 Brookpark Rd., Cleveland, OH 44135, USA; neudeck@nasa.gov; 5 Department of Electrical and Photonics Engineering, Technical University of Denmark, Ørsteds Plads, Building 343, DK-2800 Kgs. Lyngby, Denmark; haou@fotonik.dtu.dk; 6 CNR-IMM, 40129 Bologna, Italy; roncaglia@bo.imm.cnr.it; 7 Electrical Engineering Department, University of South Florida, 4202 E. Fowler Avenue, ENG 030, Tampa, FL 33620, USA; saddow@usf.edu; 8 INFN-LNS, Via Santa Sofia 62, 95124 Catania, Italy; tudisco@lns.infn.it

**Keywords:** silicon carbide, high temperature devices, detectors, photonics, MEMS, biomedical devices

## Abstract

In recent years, several new applications of SiC (both 4H and 3C polytypes) have been proposed in different papers. In this review, several of these emerging applications have been reported to show the development status, the main problems to be solved and the outlooks for these new devices. The use of SiC for high temperature applications in space, high temperature CMOS, high radiation hard detectors, new optical devices, high frequency MEMS, new devices with integrated 2D materials and biosensors have been extensively reviewed in this paper. The development of these new applications, at least for the 4H-SiC ones, has been favored by the strong improvement in SiC technology and in the material quality and price, due to the increasing market for power devices. However, at the same time, these new applications need the development of new processes and the improvement of material properties (high temperature packages, channel mobility and threshold voltage instability improvement, thick epitaxial layers, low defects, long carrier lifetime, low epitaxial doping). Instead, in the case of 3C-SiC applications, several new projects have developed material processes to obtain more performing MEMS, photonics and biomedical devices. Despite the good performance of these devices and the potential market, the further development of the material and of the specific processes and the lack of several SiC foundries for these applications are limiting further development in these fields.

## 1. Introduction

Newly emerging semiconductors, such as silicon carbide (SiC), are attractive for advanced power devices [1,2,3,4,5,6] due to their superior physical properties. Owing to the remarkable improvement in SiC wafer quality and the progress in device technology, high-voltage SiC Schottky barrier diodes (SBDs) and field-effect transistors (FETs), which significantly outperform Si counterparts, have been demonstrated and, in recent years, the market of SiC power devices has increased considerably thanks to the application of electric vehicles.

In recent years, several interesting applications of this material in different fields (MEMS, optical devices, radiation detectors, biomedical devices, quantum devices, high temperature electronics, photovoltaics, water splitting, etc.) [7,8,9,10] have been proposed thanks to the outstanding mechanical, optical, and radiation hardness properties, as well as the biocompatibility properties of this material.

This wide bandgap semiconductor exists in nature in several crystalline structures called polytypes, that are differentiated by the stacking sequence of the tetrahedrally bonded Si-C bilayers. Through variations in this stacking sequence, SiC adopts different atomic arrangements and symmetries, from hexagonal to cubic and rhombohedral; each has a different set of physical properties.

Today, the 4H form of SiC grabs the headlines due to the ramping volumes of diodes and MOSFETs for the electric vehicle market. However, despite all this success, it is not the best polytype in many important regards. In fact, for several applications such as MEMS, optical devices and biomedical devices, where a thin layer is needed or it is necessary to realize a suspended structure, the 3C-SiC polytype can be interesting because 3C-SiC can be grown epitaxially on a silicon substrate [11], which is easier to etch silicon into to realize the suspended structure. Furthermore, the cost of the silicon substrate is much lower with respect to the 4H-SiC substrate. Then, in this review we will discuss new applications of silicon carbide using both 3C-SiC and 4H-SiC polytypes.

In Table 1, all the main properties of these polytypes are reported and compared with silicon. Then, in the rest of the introduction, we will discuss the main advantages of silicon carbide for a selected numbers of applications and, in the results section, the same applications will be described in particular, and the main results reviewed. 

SiC electron devices are attractive building blocks for integrated circuits (ICs) operating in harsh environments, such as with high temperature or particle radiation. Several logic circuits, amplifiers, and sensors have already been extensively developed by using (i) a combination of a SiC n-channel JFET and a resistor [12] and (ii) SiC npn bipolar junction transistors (BJTs) [13]. Although these studies have demonstrated very promising results, including stable operations at very high temperatures (>500 °C), one drawback of these devices is their relatively high power consumption, which is inherent to the device structures. In Si LSIs, a Complementary MOS (CMOS) configuration has been almost exclusively employed because of the negligibly small static power consumption and easy scaling [14]. Therefore, it is naturally expected that SiC-based CMOS devices can take a key role in harsh environment ICs, if the technology becomes mature and reliable in these application environments.

SiC presents several advantages with respect to silicon for detector applications. The higher band-gap (Table 1) produces both a lower leakage current of the junctions (with a lower signal/noise ratio) and a detector that is blind to visible light. Furthermore, the defects produced by high level radiation produce a lower leakage current increase with respect to a semiconductor, with a lower band-gap. From the point of view of the radiation hardness, another important property is the high displacement energy, which considerably reduces the number of defects generated from a high energy particle. The large difference between the electron and the hole mobility (Table 1) gives the opportunity to realize a charge identification using pulse shape analysis, as in the case of silicon. The high saturation velocity (Table 1) also gives the advantage, if a fast electronic is used, of realizing a fast detector that can be used with high particles flux with a low dead time. The only disadvantage with respect to silicon detectors is that the energy used to create an electron/hole pair is double in SiC (Table 1), and that the signal produced is half of the signal produced in silicon from the same particle. This disadvantage is compensated by the low leakage current and the good signal/noise ratio.

Even new device structures such as SiC Nano Wires (NWs) have been tested on both 4H-SiC [15] and 3C-SiC [16] to realize new FET devices or UV [17] or gas sensors [18].

An optical chip is deemed to provide a solution to overcome the bottleneck encountered in the integrated circuits, where Moore’s Law is interrupted by the line width approaching the physical limit of atoms. In this battle, a lot of material platforms are being explored, such as silicon, silicon dioxide, silicon oxynitride, silicon nitride, InP, GaAs, diamond, AlN, lithium niobate, etc. 

SiC is emerging as a material platform for photonics integrated circuits (PICs) [19] and quantum photonic integrated circuits (QPICs) [20] because it possesses unique photonic properties. Compared to other material platforms, such as III-V semiconductors, diamond, etc., photonic applications of SiC leverage the mature material growth and processing developed for SiC power electronics. Compared to Si, as shown in Table 1, SiC has a wider bandgap, which not only means that SiC has a wide transmission window from near ultraviolet (UV) to mid-infrared, but also means that serious two-photon absorption at the telecommunication wavelength in SiC could be ignored in SiC; SiC has a higher second-order nonlinear susceptibility, which could provide efficient second-harmonics generation and electro-optical modulation. SiC has a rather high refractive index, which enables tight light confinement and assures strong light–SiC interaction. SiC also has a reasonable nonlinear refractive index, which is important to demonstrate wavelength conversion through four-wave mixing.

Silicon Carbide is a suitable material for fabricating even MEMS devices [21,22,23,24,25,26,27,28], particularly when it is grown on silicon substrates, because in this case it is relatively easy to create suspended structures by removing the underlying silicon with various consolidated techniques. Moreover, the mechanical properties of epitaxially grown SiC on silicon are excellent (Table 1), and this allows MEMS devices with very good performances to be obtained. Quite often, such devices are investigated for harsh environment applications in which the well-known properties of SiC related to corrosion and high-temperature resistance can be fully exploited.

SiC has a long history as a chemically robust material. Indeed, the inability to etch SiC using wet chemistry has made the fabrication of devices more challenging and expensive. However, it is this inherent ‘robust chemical resistivity’ material property that makes SiC attractive for in vivo biomedical applications [29]. The human body is often referred to as a harsh environment. Any device interacting within this environment is constantly exposed to a salinated, ionic environment full of proteins which “foul”, or coat, its surface. Additionally, the body’s own active inflammatory system rapidly identifies foreign objects and activates to unleash various physical and chemical attacks, complete with oxidizers, in order to eliminate or dissolve the foreign intruder. A failure to eliminate the intruder will lead to the ‘walling off’ or otherwise separating of the device, shutting off its access to the bodily environment. A biomedical device fabricated from materials that do not succumb to inflammatory attacks and do not telegraph their presence by shedding ions to further alert the immune system is key to overall device functionality. The robust chemical nature of SiC has demonstrated, through many examples, a minimal to unmeasurable immune system response when applied in chronic implantations [30,31].

This review has been divided into different sections, whereby the different aspects of these new SiC applications have been reviewed and whereby the actual status of the technology has been explained in detail. In the final conclusion and outlook section, the final summary and outlook of the different applications are reported.

## 2. Results and Discussions

### 2.1. High Temperature SiC Devices for Aerospace Applications

The ability of SiC semiconductors to offer important electrical functionality at extreme high temperatures (well beyond the roughly 250 °C effective temperature ceiling of silicon semiconductor electronics) was a recognized motivation of the early US Government sponsorship of foundational SiC electronic materials research and development in the 1980s. The anticipated benefits to aerospace systems were reflected by the fact that aerospace research agencies (including NASA, the US Air Force, and the US Navy) sponsored the majority of the early SiC electronics research. However, as SiC technology advanced through early-stage commercialization in the 1990s, high temperature aerospace motivations were overtaken as SiC high-power devices’ benefits at conventional temperatures became recognized as far more lucrative and more rapidly obtainable [32]. As such, the intervening decades have witnessed development, mass-production, and the increasingly widespread infusion of SiC-based power electronics playing an increasingly critical role in the worldwide drive to implement energy-efficient electric systems, helping to reduce greenhouse gas emissions responsible for global climate change, whereas high-temperature SiC device development has comparatively languished. 

With SiC power electronics manufacturing now firmly established and expanding on a global scale, there has been some resurgence of interest in leveraging a fraction of this foundry capacity towards realizing SiC extreme environment electronics. However, further technology maturation investment is necessary to reliably extend the upper temperature limits of the entire SiC electronics manufacturing chain, including device- and system-level packaging. This section briefly surveys some of the motivating applications for which small quantities of high temperature SiC electronics, after becoming reliable and available, are expected have a high-value impact on aerospace system performance. 

**Figure 1:**
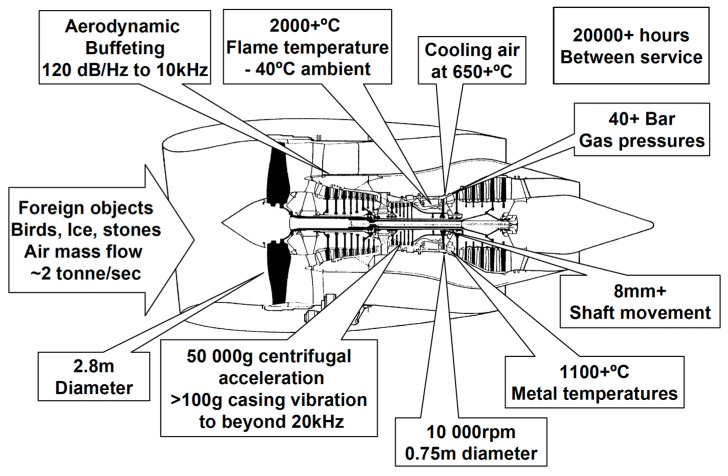
Environmental challenges of the jet engine environment [33].

Complex electronics and sensors are increasingly relied on to enhance the capabilities and efficiency of modern atmospheric flight vehicles, including military and commercial jet aircraft. Some of these electronics and sensors monitor and control vital engine components and aerosurfaces that operate at high temperatures, but these presently must reside in environmentally controlled areas of far lower temperature. This necessitates either (1) the use of wire runs between the sheltered electronics and the hot-area sensors and actuators, or (2) active fan-air or liquid (sometimes fuel) cooling of the electronics/sensors located in high-temperature areas. These low-temperature-electronics approaches suffer from significant drawbacks of added weight, poorer system reliability, increased maintenance and repair, and the degradation of sensing fidelity. A family of extreme temperature SiC electronics and sensors that could reliably function in hot areas of aerospace vehicles would alleviate the above-mentioned technical obstacles to enable substantial performance gains. In the case of jet engines, future performance-enhancing active and distributed electronic engine controls [33] become more attainable when SiC devices handle higher temperature zones closer to key points of sensing and combustion/flow control, working in concert with silicon electronics residing in lower temperature engine areas. Figure 1 illustrates the additional environmental demands that such sensors/electronics could encounter in the jet engine operational environment, in addition to high temperature. 

There is also an increasing drive to electrify aircraft, both in terms of replacing primary flight control hydraulics with more compact and lower-maintenance electromechanical actuation [34,35], and in electrifying aircraft propulsion towards global de-carbonization [36]. While conventional-temperature SiC power devices are already improving developmental aircraft electric power systems, pushing SiC power devices to higher reliable operating temperatures opens the possibility of systems with substantially less thermal management overhead. For the most part, similar benefits of SiC high temperature operation also apply to spacecraft power systems. Space nuclear power is expected to play a key role in the advanced exploration of the outer solar system, and these future power systems will require control and monitoring circuits for safe and optimum reactor performance [37]. The use of heat-tolerant, radiation-hardened SiC circuits will greatly reduce the shielding needed to protect the reactor control electronics and enable closer-proximity placement, both of which should trim considerable weight from the power system.

For spacecraft operating near the Sun, SiC extreme environment electronics would enable significant reductions in spacecraft shielding and heat dissipation hardware, so that more scientific instruments could be included on each probe. Nowhere is the scientific exploration mission gain offered by the SiC extreme environment electronics larger than for robotic landers to the explore the 460 °C, 9.3 MPa surface of Venus. As of the time of writing, the longest duration of science data return from the surface of Venus surface is 2 h and 7 min by the Venera 13 lander, whose ~760 kg mass was dominated by a massive pressure vessel needed to briefly protect its electronics from the Venus atmosphere [38].

**Figure 2:**
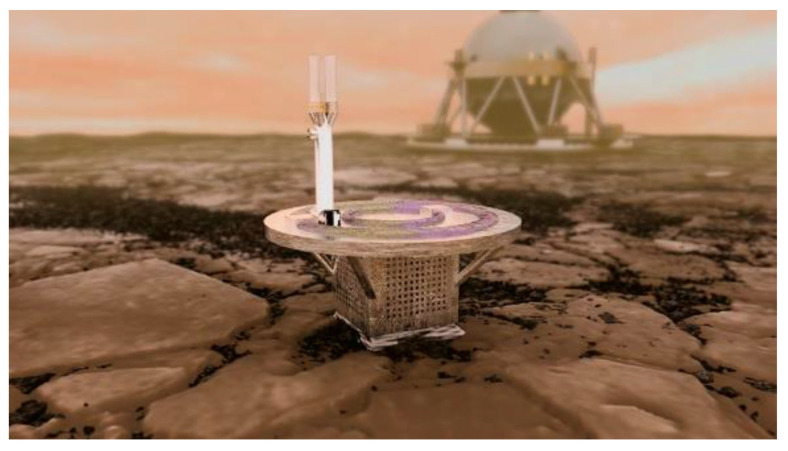
Concept of ~10 kg LLISSE lander conducting a 60-day mission on Venus enabled by SiC electronics operation unprotected from the 460 °C 9.3 MPa surface environmental conditions. In the background is a protected-electronics lander design weighing over 500 kg whose mission duration is less than 1 day [40].

Within recent years, however, SiC-integrated circuits prototyped by the NASA Glenn Research Center have demonstrated operation up to 1440 h (60 Earth days, whose duration was limited by test chamber scheduling) directly exposed to (without any form of protection) high-fidelity test-chamber reproductions of the Venus surface environment (same temperature, pressure, and chemical composition) [39]. Based upon these landmark demonstrations, NASA has now initiated the development of paradigm-shifting Venus surface mission concepts and demonstration hardware. An example of such a mission concept is the Long-Lived (or Long-Life) In Situ Solar System Explorer (LLISSE, Figure 2) lander, weighing ~10 kg but capable of transmitting Venus’ surface meteorological data (including temperature, pressure, wind, and concentrations of selected atmospheric gasses) for at least 60 Earth days [40]. In other words, the Venus durability of SiC electronics would critically enable a roughly 70-fold reduction in lander mass while simultaneously enabling a nearly 700-fold lengthening of the robotic Venus surface mission duration.

For some applications, the integration of both low-power (for sensing and control) and high-power electronics (for actuation) is necessary. It is important to note that T ≥ 400 °C SiC integrated circuits are unlikely to achieve chip complexities, operating frequencies, and low power consumption approaching modern room-temperature silicon CMOS chips. Nevertheless, SiC-integrated circuit chips of sufficient complexity to satisfy crucial extreme-temperature electronic functionality are in prototype fabrication, ranging from amplification and digitization up through to a simple microprocessor [41]. These SiC prototype chips are already comparable in complexity and functionality with the 1970s silicon ICs that successfully ran Viking and Voyager robotic planetary exploration missions.

Before SiC flies in harsh environments, significant maturation, commercialization, and environmentally realistic ground testing qualification remains to be conducted. Fight qualification demands large safety margins and comprehensive statistical testing that ensures reliable operations for far longer than the actual mission duration. Additional applications that would benefit from reliable extreme temperature SiC electronics include deep-well drilling and automotive and industrial process control applications [42]. The high temperature market will always be small compared to the conventional temperature electronics market. The compatibility and leveraging of existing SiC foundry manufacturing is considered vital to the establishment of a sustainable SiC-based electronics ecosystem necessary to realize widespread reliable deployment into extreme environment aerospace systems. 

### 2.2. SiC Complementary MOSFETs and JFETs

#### 2.2.1. Features and Basic Configurations of SiC Complementary FETs

As described in the Introduction section, extremely low static power consumption is a key advantage of complementary FETs for IC applications. Complementary FETs can be easily fabricated with SiC when compared with other wide bandgap semiconductors, because selective n- and p-type doping in a wide range is feasible via ion implantation in SiC. The commercialization of high-voltage SiC power MOSFETs also serves as a tail wind for the development of SiC CMOS, since the SiC MOS technology, at least for the n-channel devices, has reached production level despite a high density of interface defects. 

**Figure 3:**
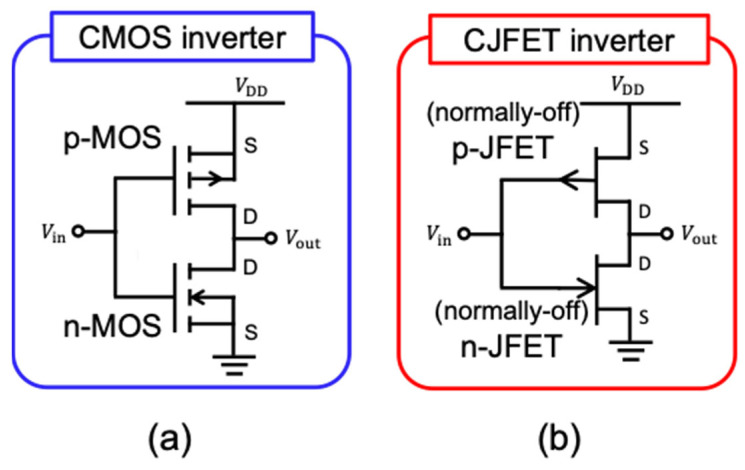
Circuit diagrams of (**a**) CMOS and (**b**) CJFET inverters.

Figure 3 shows the circuit diagrams of (a) CMOS and (b) CJFET (Complementary JFET) inverters. In either case, it is essential that (i) the drain current of the n-channel FET is well balanced with that of the p-channel FET, (ii) both the n- and p-channel FETs must be normally-off, and (iii) the absolute values of the threshold voltage for n- and p-channel FETs must be very similar (ideally identical) to each other. In this section, the development of SiC CMOS and CJFET devices are briefly reviewed and technological challenges are discussed.

#### 2.2.2. SiC CMOS

Most technology of Si CMOS, such as basic device structures, device isolation, and device layout for circuit construction, can be transferred to SiC CMOS. However, the development of SiC p-channel MOSFETs has been much behind compared with SiC n-channel MOSFETs, which have been extensively investigated for power MOSFET fabrication. Other challenges in SiC CMOS include the lack of a self-alignment process for the source/drain formation through a poly Si gate, which results in large parasitic capacitances in SiC MOSFETs. A high density of interface states and oxide traps affects the performance (channel mobility, transconductance) and reliability of SiC MOS devices [43,44]. For example, the channel mobility of SiC p-channel MOSFETs is typically 10–14 cm^2^/Vs on a lightly doped n-body, and decreases to 4–6 cm^2^/Vs on a relatively heavily-doped body [45,46]. Nevertheless, various SiC CMOS ICs have successfully been demonstrated [47,48,49,50,51,52,53,54,55].

**Figure 4:**
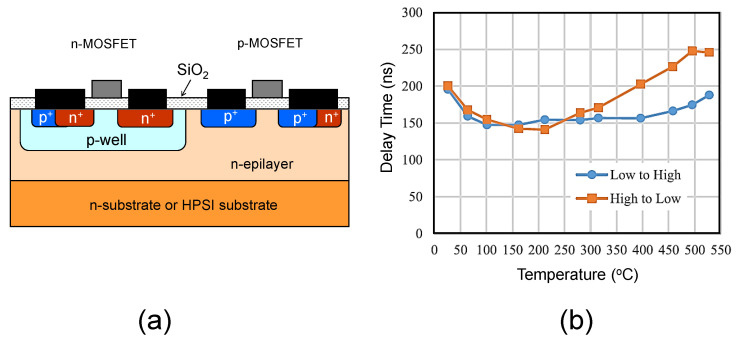
(**a**) Schematic structure of a SiC CMOS inverter and (**b**) propagation delay of a gate driver constructed with SiC CMOS as a function of temperature [49].

Figure 4 depicts (a) the schematic structure of a SiC CMOS inverter, and (b) the propagation delay of a gate driver constructed with SiC CMOS as a function of temperature [49]. The gate oxide thickness and the channel length of these MOSFETs were 40 nm and 1.2 μm, respectively, and the channel width of the p-channel MOSFETs was five times larger than that of the n-channel MOSFETs to satisfy the current balance. This gate driver was designed to work at 500 kHz, with a supply voltage of 15 V, and exhibited good operation at a very high temperature of over 500 °C. The 540 °C operation of voltage-reference and current-reference circuits with SiC CMOS has been also reported [50]. Other than high-temperature operation, a very high resistance against γ-ray irradiation over 1 MGy was achieved for SiC CMOS-based transimpedance amplifiers for nuclear plant applications [51], which is surprising because it was presumed that resistance against irradiation is limited by the gate oxide damage rather than SiC bulk defects. Another interesting approach is the fabrication of a multi-pixel UV image sensor integrated with a readout circuit in SiC CMOS technology [52]. Owing to the wide bandgap of SiC, the sensor is inherently “solar blind” and the sensitive spectral bandwidth of the UV detector is much narrower than the Si-based UV detector, which makes this device attractive for flame detection, healthcare, etc. In addition, monolithic SiC power ICs, which consist of a high-voltage SiC power MOSFET and SiC CMOS logic circuits, have recently been demonstrated [53,54,56,57]. 

#### 2.2.3. SiC CJFET

In CJFET, the n- and p-channel MOSFETs in CMOS are replaced with n- and p-channel JFETs, respectively, as shown in Figure 3b. In CJFET ICs, well-established CMOS’ circuit architecture can be basically used, whereas the normally-off operation of both the n- and p-channel JFETs is mandatorily required. The CJFET concept was proposed in GaAs technology in the 1980s [58]. However, the gate voltage and supply voltage are limited below approximately 1 V due to the relatively small bandgap of GaAs, because the excessive gate current flows when the gate-channel pn junction is forward biased. In SiC CJFET, the gate and supply voltages can be higher, up to 2.0–2.5 V due to its wide bandgap, enabling larger flexibility in the IC design and operation compared with GaAs or Si CJFET technology. The simulation of SiC CJFET with promising results was reported [59].

**Figure 5:**
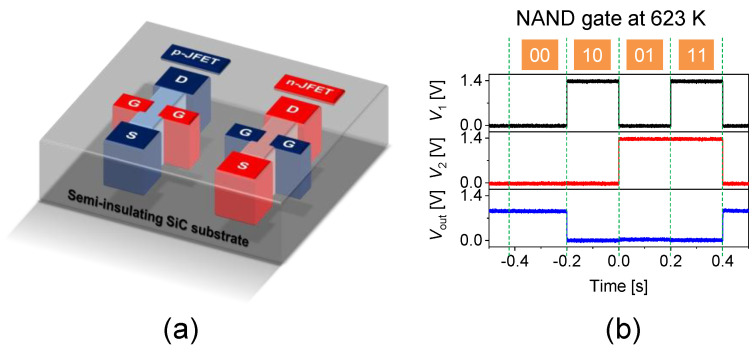
(**a**) Schematic structure of a SiC CJFET and (**b**) characteristics of a NAND gate at 350 °C fabricated with SiC CJFET [61].

The fabrication of CJFET devices by epitaxial growth is rather difficult, since both n- and p-channel JFETs must be constructed on a common (same) substrate. Thus, ion implantation is an essential process for the fabrication of CJFET ICs [60]. Figure 5 shows (a) the schematic structure of a SiC CJFET, and (b) the characteristics of a NAND gate at 350 °C fabricated with SiC CJFET [61]. In this approach, double-gate CJFETs were fully fabricated by P^+^ and Al^+^ implantation into a high-purity semi-insulating (HPSI) SiC substrate, the resistivity of which exceeded 10^12^ Ωcm at room temperature. Since the Fermi level of an HPSI SiC substrate is located near the midgap, sufficiently large potential barriers are expected at the n-SiC/HPSI-SiC and p-SiC/HPSI-SiC interfaces. The gate length and the channel doping density were 4 μm and about 5 × 10^16^ cm^−3^, respectively. The threshold voltage was 0.74 V and −0.78 V for n- and p-channel JFETs, respectively, indicating almost symmetric and normally-off characteristics. The NAND gate with a supply voltage of 1.4 V exhibited a stable operation at 350 °C. SiC CJFET ICs are still in the very early stage, and further studies are required.

#### 2.2.4. Technological Challenges of SiC CMOS and CJFET

Both SiC CMOS and CJFET ICs face several technological issues which need to be resolved. Major advantages and concerns regarding SiC CMOS and CJFET are summarized in Table 2. SiC CMOS ICs are attractive for most applications, but performance enhancement and the improvement of oxide-related reliability are key remaining issues. On the other hand, SiC CJFET ICs will be promising for low-voltage (~2 V) ICs under very harsh environments such as very high temperatures and heavy radiation. These technologies will find suitable applications because of low power consumption, though normally-on SiC JFET and BJT technologies [12,13] are more advanced at present.

The main performance-limiting factors in SiC CMOS, such as very low p-channel mobility, originate from the defects present at and near the MOS interface. Since an about two-fold improvement in p-channel mobility (and also in n-channel mobility) is achieved on (11–20) [62], a FinFET structure with the nonpolar face on the sidewalls may be promising for SiC CMOS. Another severe concern with SiC CMOS is the threshold voltage instability [63], and this problem seems to be more stringent for p-channel MOSFETs (negative bias-induced threshold voltage instability) [64]. Although more aggressive scaling will be required to increase the switching frequency in the future, short-channel effects must be avoided via appropriate device designing [65].

On the other hand, the threshold voltage of JFETs is determined by the channel doping and thickness, and is not affected by the traps near the MOS interface. The threshold voltage shift at elevated temperature is easily predicted by simple calculation. However, SiC CJFET technology is not mature, and the JFET structure itself is still the subject of study in pursuit of superior control of the threshold voltage. A guideline for avoiding the short-channel effects has recently been reported [66]. 

It should be noted that both SiC CMOS and CJFET possess several common technical issues which need to be improved. Such technical issues include the formation of low-resistivity p^+^ regions via ion implantation and low-contact resistivity ohmic contacts on p^+^ regions. In SiC CMOS and CJFET, the active regions are basically formed by ion implantation. Ion implantation into SiC induces the generation of various points and extended defects. Most of these defects create deep levels, and it is known that many of these deep levels survive post-implantation annealing at 1650–1800 °C [67,68,69,70]. These deep levels work as carrier traps, leading to the relatively higher resistivity of the ion-implanted regions than that of epitaxial layers at a given doping density. Pre-existing deep levels in an HPSI SiC substrate [71] also affect the electrical properties of n- and p-type regions formed by ion implantation into the HPSI substrate. The reduction of these defects is another important challenge for high-performance SiC CMOS and CJFET devices. Furthermore, the reliability of contacts and dielectric films at very high temperatures should be carefully investigated. In addition, the SPICE simulation of these SiC ICs, especially CMOS ICs, must be established to accelerate the IC development [53].

## 3. SiC Detectors

The use of a solid medium is of great advantage in many radiation detection applications. Semiconductor-Detectors (SD) are sensitive to many kinds of ionizing radiation, and their pulse height spectra can be used also to measure the particles’ incident energy. 

The SD performances (energy resolution, noise, response time, etc.) are affected by the internal processes within the active volume, as well as by the readout techniques. Energy resolution depends essentially on the statistical fluctuations of electron-hole pairs created inside by ionizing radiation and by the others’ detector-related noise sources. Under the assumption of high detector resistivity, the major noise sources are the thermal fluctuation of carriers within the detector active region and the leakage current across the detector electrodes. Normally, a lower temperature helps to reduce these contributions. However, new wide band gap materials may be useful to prototype low noise detectors working at room temperature.

A new challenge for detector manufacturing is represented by Silicon-Carbide (SiC) material. The frontier activities on nuclear and subnuclear physics and applications require devices with excellent performance in terms of high flux radiation operation. The wide band-gap of SiC determines the main advantage over Silicon (Si) devices, and the extremely low leakage current density of a SiC versus Si device is about $J_{pn}^{SiC}/J_{pn}^{Si}≃10^{-3}$. 

Wide-band gap materials are also extremely appealing for their expected features in terms of radiation hardness. A high flux in radiation is known to cause an appreciable deterioration in the detector performance [72,73,74,75,76,77]. The primary evidence of radiation damage is a fluence-proportional increase in the leakage current, resulting in a loss of energy resolution and charge collection efficiency.

The needs of the new and ambitious scientific projects of the Italian National Institute of Nuclear Physics (INFN) and the intensive collaboration with the Italian Institute for Microelectronic and Microsystem CNR-IMM have recently generated new and innovative technologies to produce SIC detectors.

This activity, named SiCILIA, was totally funded by the INFN to manufacture prototypes of SiC radiation hard detectors with the following geometrical prescriptions: (i) relatively large detection area (≥1 cm^2^) devices; (ii) thicknesses in the range of 50–300 µm; and (iii) devices without dead-layers on both sides (front and back) in order to guarantee the possible implementations of more complex detection units (e.g., the telescope configurations). At the beginning of the SiCILIA project, the main limitations were the thicknesses, the active area and manufacturing technologies. Most of the SiC devices were produced through Schottky junction’s implementation. Regarding this topic, one of the first results of SiCILIA R&D was the prototyping of new p-n junction device. 

**Figure 6:**
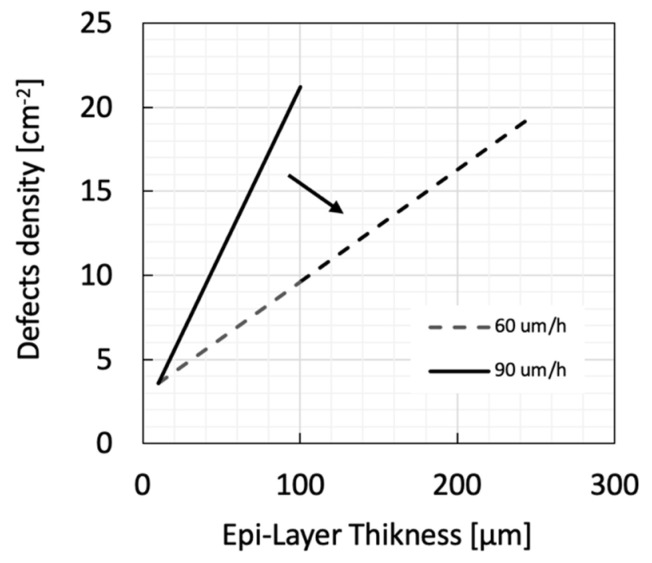
Electrical defect density vs. epi layer thickness for two different high-growth-rate processes. A considerable improvement in the defects’ density has been observed at the lower growth rate.

**Figure 7:**
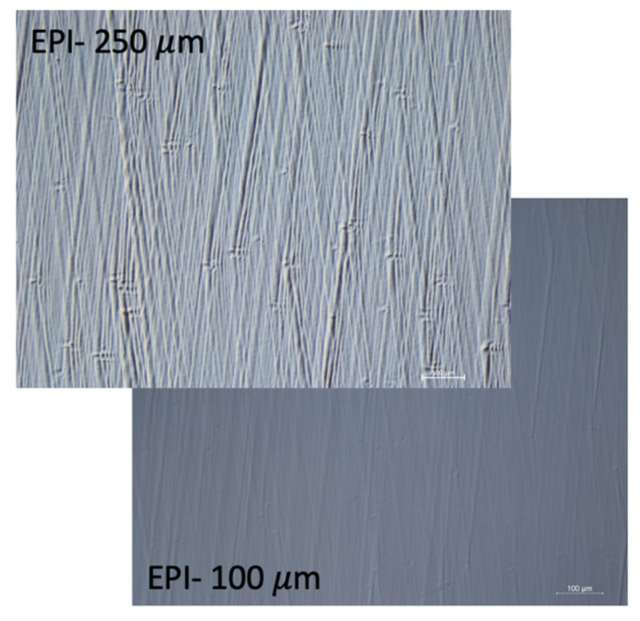
Optical microscope images of two different epitaxial layer thicknesses. It is possible to observe an increase in the roughness, increasing the thickness.

The defect concentration of the N-doped epitaxial layer is a further critical parameter for the prototyping of large area devices; it has a direct impact on the number of working devices, and then on the production yield. Furthermore, growing the epi-layer thickness increases the number of defects. One way to control this parameter is to act on the epitaxial growth rate of the CVD process [78]. In Figure 6, the trends of defects concentration as a function of layer thickness for the growth rates of 90 μm/h and 60 μm/h are shown. The reduction in the grow rate minimizes the number of defects, and good production yields have been obtained for 1.5 × 1.5 cm^2^ devices. Moreover, with this prescription we have been able to push the record for N-doped epitaxial layer thickness on a 4″ wafer up to 250 μm (see Figure 7). 

**Figure 8:**
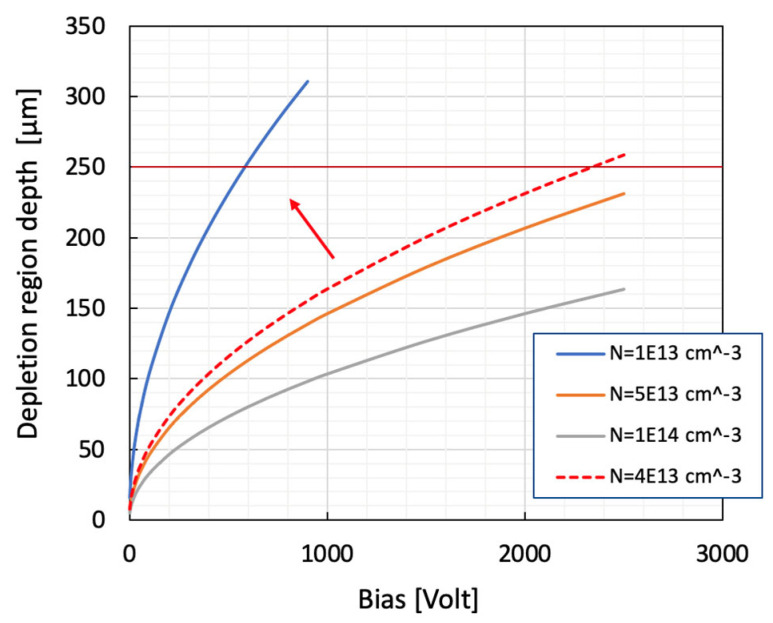
Depletion region vs. the reverse bias of a p/n junction for different epitaxial layer doping.

A very thick detector also requires a larger bias voltage to reach its working condition (full depletion). This aspect is dominated by the precision of dopants’ concentration, which is an extremely critical parameter. In the case of Nitrogen, for the N-type doping layer, the concentrations request, the maintenance of the depletion-voltage at reasonable levels, is very close to the background values. A lot of technological effort, in recent years, has been conducted to reduce the amount of background impurity inside the epitaxial reactors. New CVD-reactor architectures have been proposed [79]. Figure 8 shows the evolution of the depletion region depth as a function of reverse bias for several doping concentrations. Commercial epitaxial layer providers are not able to ensure layers, Nitrogen doped, with concentrations under N = 10^14^ cm^−3^. In this respect, the R&D activities conducted by SiCILIA collaboration have brought down the Nitrogen concentration level for a 100 um thick epi-layer on a 6″ wafer up to N = 4 × 10^13^ cm^−3^. This new record allows the full depletion condition for a 250 μm thick detector with a bias voltage under the 2.5 kV to be reached. The next goal, as is shown in Figure 8, will be to reach N = 10^−13^ cm^−3^ with a depletion voltage of a few hundred of volts in these very thick layers. 

Another important aspect for SiC detectors is to have a very low point defects concentration. In fact, to collect all the charges produced in the thick epitaxial layer, a long diffusion length and then a long carrier lifetime are necessary. For this reason, an optimization of both the epitaxial layer process and the subsequent processes to reduce the point defects inside the epitaxial layer are needed [80].

The main performances of SiCILIA detectors are listed below:

*Energy resolution *≅ 0.2% [Sicilia] for the 5.4 MeV alpha particles of the ^241^Am radioactive source. Such an estimation was extracted by using a standard nuclear spectroscopic electronic chain. This resolution can even be improved using an optimized electronic chain. 

*Time resolution *≅ 200 ps, at the limit of the used preamplifier (Ascom). Such an estimation was extracted by using a ^58^Ni beam at 70 MeV [81].

*Radiation hardness neutrons resistance*. The device was irradiated by using a 14 MeV neutrons beam, up to a total fluence of 4.45 × 10^11^ n/cm^2^. No instabilities were observed [82,83].

*Radiation hardness photons resistance.* The device was irradiated by using a 12.4 KeV photons beam up to 45 GGy without observing instability or deteriorations [84].

*Radiation hardness protons resistance.* The device was irradiated by using a 60 MeV protons beam up to 2 kGy without observing instability or deteriorations [85].

*Radiation hardness heavy ions resistance.* The device was irradiated by using a 12.5 MeV ^16^O beam and a 24.2 MeV ^27^Al beam [81]. The device was irradiated up to 10^12^ ions/cm^2^, and a reduction in the total charge collection efficiency at about 60 % was observed without a major change in the resolution. 

*Radiation hardness electrons resistance.* The device was irradiated by using a 5 MeV electrons beam, up to a total fluence of 10^13^ e/cm^2^. No instabilities or deteriorations were observed. 

Prototypes have also been tested in telescopic configurations, obtaining a good element identification (at least up to Z = 20) and isotopic identification up to Z = 14 [86]. Pulse shape analysis has been applied to the signals produced by the 100 μm thick SiC detector, obtaining promising results. Good element identification and even some isotopic identification for light charged particles has been obtained.

The future perspectives of this activity are aimed at the research, development, and manufacture of innovative medical devices.

## 4. 2D Materials’ Integration on Silicon Carbide

Two-dimensional (2D) materials have been the object of wide scientific interest during the last two decades, due to their unique electrical, optical, mechanical, and chemical properties. Their integration materials with bulk semiconductors are currently explored as an approach to enable novel device concepts, as well as to enhance the performances of state-of-the-art electronic/optoelectronic devices and sensors [87,88,89]. In particular, the hexagonal polytypes of silicon carbide (6H- and 4H-SiC) have been shown to be suitable substrates for the epitaxial growth of 2D materials with hexagonal lattice, such as graphene [90], molybdenum disulfide (MoS_2_) [91] and h-BN [92]. The resulting heterostructures allow the expansion of the range of applications of SiC beyond the field of power electronics, including RF transistors, quantum metrology and environmental, chemical and biological sensors.

The controlled graphitization of SiC during high temperature thermal processes [93,94,95] was one of the first approaches used to obtain high quality epitaxial graphene (Epi-Gr) directly on a semiconductor or semi-insulating substrate, i.e., ready for electronic devices fabrication. To date, this approach has been widely investigated on the hexagonal (4H- and 6H-) polytypes of SiC. Growth on the cubic (3C) polytype has also been explored [96], in view of the possibility of integrating graphene with 3C-SiC layers on larger area and cheaper silicon wafers. However, the material quality of heteroepitaxial 3C-SiC, with a high density of conducting defects [97], currently limits the possibility of exploiting the properties of graphene grown on its surface in electronics. For graphene grown on hexagonal SiC substrates, the structural properties (number of graphene layers, and atomic configuration of the interface with SiC) and electronic properties (doping, carrier mobility) have been shown to be critically dependent on the graphitization conditions (i.e., the substrate temperature and pressure in the process chamber), and on the crystal orientations and surface morphology [98]. Very different growth results have been obtained on the Si face (0001) [99], the C face (000-1) [100], and the low index (11–20) and (1–100) non-polar faces [101,102]. For on-axis SiC(0001), high temperature (>1600 °C) thermal decomposition in inert gas (Ar) at atmospheric pressure typically results in the formation of highly uniform monolayer (1L) graphene rotationally aligned to the substrate, with bilayer (2L) or trilayer (3L) patches localized at SiC step edges. Multilayers of graphene with Bernal stacking and epitaxially aligned with the substrate are obtained on 8° or 4° off-axis SiC(0001) [103,104]. On the other hand, the growth on the (000-1) face and non-polar faces is difficult to control and yields a laterally inhomogeneous distribution of graphene multilayers, in which the graphene layers are reciprocally misoriented, and it lacks an epitaxial relation with the substrate [105,106]. The excellent uniformity of Epi-Gr on the (0001) SiC orientation is related to the peculiar growth mechanism mediated by the formation of a carbon buffer layer (BL) covalently bonded to the Si face [107]. Furthermore, the presence of the BL between the SiC substrate and graphene is responsible for the peculiar electronic properties of this heterostructure, such as the high n-type doping (10^13^ cm^−2^) of Epi-Gr (induced by the high density of positively charged Si dangling bonds at BL/SiC interface) and the Ohmic-like behavior of the Epi-Gr/SiC(0001) contact [108]. One of the most appealing features of this Epi-Gr/SiC material system is the possibility of tuning both the graphene doping and the vertical current injection at the heterointerface through the controlled intercalation of different kinds atomic species [109]. As an example, hydrogen intercalation proved to be very effective to break the covalent bonds between the BL and SiC(0001) and to saturate Si dangling bonds, thus transforming the BL in a lowly doped quasi-free-standing Epi-Gr layer [110] featuring a rectifying contact to SiC [111].

From the application viewpoint, Epi-Gr growth provides SiC with a two-dimensional electron gas (2DEG) of Dirac fermions. The high mobility (~1000 cm^2^V^−1^s^−1^) of this 2DEG has been exploited to demonstrate RF transistors, with a cut-off frequency exceeding 100 GHz [112,113] integrated on semi-insulating SiC wafers. Furthermore, Epi-Gr on SiC has been employed to demonstrate quantum Hall effect (QHE) resistance standards [114] operating under more relaxed conditions (lower magnetic fields, higher temperatures) than the traditional QHE devices based on AlGaAs/GaAs, thus leading to strong advancements in metrology [115]. The strong effects of the interaction with external gas or liquid species on Epi-Gr transport properties resulted in the demonstration of environmental sensors with outstanding sensitivity [116,117], although optimizing selectivity still remains a challenge. Proper surface functionalization strategies of Epi-Gr also allowed the demonstration of biosensors targeted for the point-of-care and label-free diagnostics of several kind of diseases [118,119]. 

**Figure 9:**
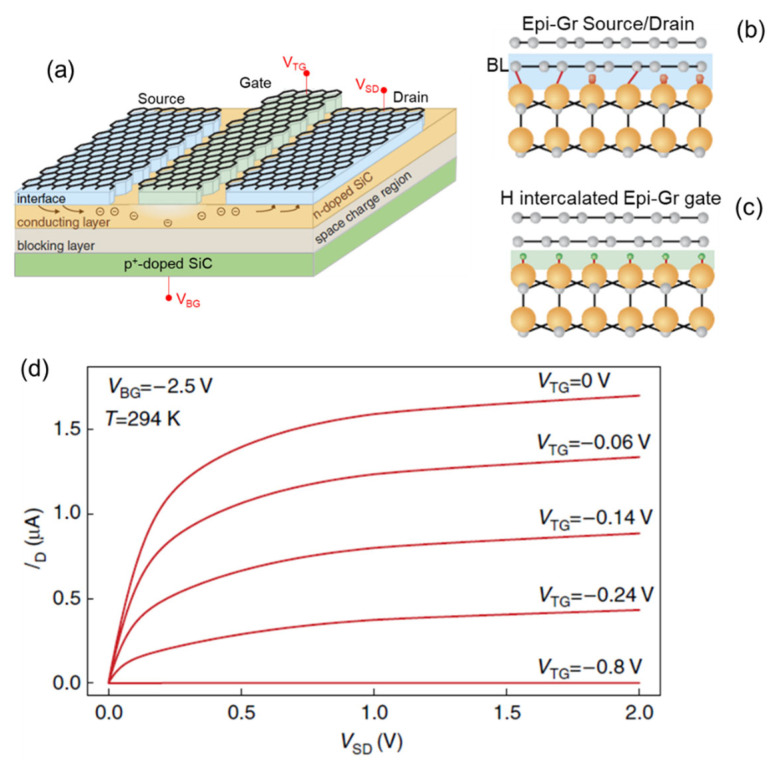
(**a**) Schematic of the transistor structure with an n-type (~10^15^ cm^−3^) epitaxial 6H-SiC channel on top of p+-SiC (~2 ×10^18^ cm^−3^), where the source/drain Ohmic contacts and the gate Schottky contact were all obtained with Epi-Gr, by properly tailoring the Epi-Gr/SiC interface properties with selective area hydrogen intercalation (**b**,**c**). (**d**) Output characteristics of the transistor, measured at T = 294 K, for fixed negative back gate bias and different top-gate biases. Images adapted from Ref. [111].

In most of the above-mentioned applications, silicon carbide simply worked as the substrate for Epi-Gr, i.e., it was not an active element of the device. The realization of devices exploiting the combination of SiC and graphene electronic properties, as well as the current transport through Epi-Gr/SiC interfaces, has been also proposed. As an example, taking the benefit of graphene transparency in a wide wavelength range and the wide bandgap of SiC, UV photodetectors based on Epi-Gr Schottky contacts n-type SiC have been demonstrated, showing very good responsivity and response speed [120,121]. Hertel et al. [111] proposed a transistor structure with an n-type (~10^15^ cm^−3^) epitaxial 6H-SiC channel on top of p^+^-SiC (~2 × 10^18^ cm^−3^), where the source/drain Ohmic contacts (with specific contact resistance ρ_c_ ≈ 6 × 10^−2^ Ωcm^2^) and the gate Schottky contact (with barrier height Φ_B_ ≈ 0.9 eV, evaluated by I-V analyses) were all obtained with Epi-Gr, by properly tailoring the Epi-Gr/SiC interface properties with selective area hydrogen intercalation (see schematics in Figure 9a–c). The conduction in the SiC channel was efficiently modulated by both the back gate bias (V_BG_), controlling the space charge region of the p^+^/n SiC junction, and by the top gate bias (V_TG_) applied to the Epi-Gr Schottky contact, thus allowing the device to operate as a normally-on transistor (see the output characteristics in Figure 9d) or as normally-off transistor.

In addition to the semi-metallic graphene, the integration of 2D semiconductors, such as the 2H-MoS_2_, on silicon carbide has been recently investigated. This layered 2D material is composed by the stacking of MoS_2_ layers connected by weak van der Waals bonds, while each triatomic MoS_2_ layer is formed by a plane of Mo atoms sandwiched between two planes of S atoms with covalent bonds. Noteworthily, MoS_2_ exhibits a thickness-dependent energy bandgap, i.e., E_g_ ≈ 1.2–1.3 eV (indirect bandgap) for two/three layers to multilayers of MoS_2_, which increases to E_g_ ≈ 1.8–1.9 eV (direct bandgap) for monolayer (1L) MoS_2_ [122]. These bandgap values make the combination of MoS_2_ with the WBG 4H-SiC attractive for the fabrication of photodetectors with high responsivity both to invisible and the UV spectral range [123]. Furthermore, thanks to the dangling-bonds-free nature of MoS_2_ layers, the possibility of realizing MoS_2_/4H-SiC heterojunction diodes with atomically sharp interfaces has been recently evaluated by different research groups [93,124,125]. In this respect, the epitaxial growth of semiconducting 2H-MoS_2_ on 4H-SiC is favored by the low lattice constants mismatch (~2.9%) between the two hexagonal crystals on the (0001) plane. To date, different deposition methods have been investigated for the growth of MoS_2_ on SiC, such as the chemical vapor deposition (CVD) [123] and pulsed laser deposition (PLD) [125,126]. The CVD growth of MoS_2_ is commonly carried out in a tube furnace with two different heating zones, one at a lower temperature (150–180 °C) hosting a crucible with S powder, and the second at a higher temperature (700–800 °C) hosting a crucible with MoO_3_ powders and the substrate. The precursors’ vapors emitted from the two crucibles are transported to the heated substrate by an inert carrier gas (Ar). This growth method typically results in the nucleation and growth of crystalline MoS_2_ domains with different shapes, and lateral sizes of tens of micrometers. However, achieving uniform coverage on a wafer scale represents a challenge, due to the difficulty of controlling the vapors’ flows for the two precursors, especially MoO_3_, on the entire wafer area. As an alternative approach, the sulfurization of pre-deposited Mo or MoO_x_ films on the SiC surface has been demonstrated as an effective method to obtain uniform and conformal MoS_2_ coverage on a large area. In this case, the final thickness of the MoS_2_ film can be controlled down to the monolayer level [124] by properly calibrating the initial Mo or MoO_x_ film thickness and the temperature for the sulfurization process. The sulfurization approach has been adopted by different research groups to demonstrate p^+^-MoS_2_/n^+^-4H-SiC heterojunction tunnel diodes for fast-switching applications. Lee et al. [91] obtained p^+^-doped MoS_2_ multilayers on an n^+^ 4H-SiC substrate via the sulfurization of thin Mo/Nb/Mo stacks at a high temperature (1000 °C), where Nb atoms had the role of acceptors for MoS_2_. Although degenerate p-type doping of MoS_2_ was achieved, the current injection across these p^+^ MoS_2_/n^+^ SiC heterojunctions was found to be dominated by multi-step recombination tunneling through midgap states in SiC, probably associated with interface defects resulting from the high temperature sulfurization process [91]. More recently, highly uniform 1L–2L MoS_2_ heterostructures with n^+^ 4H-SiC have been obtained by the sulfurization of pre-deposited, very thin (≈1.2 nm) Mo films at a temperature of 700 °C [124]. Current–voltage characteristics of these MoS_2_/n^+^-4H-SiC junctions showed pronounced negative differential resistance even at room temperature, which is a typical manifestation of band-to-band tunneling between degenerately p^+^-/n^+^-doped semiconductors. The degenerate p^+^-type doping of MoS_2_ was ascribed to the significant MoO_3_ content in the MoS_2_ film, demonstrated by XPS analyses.

**Figure 10:**
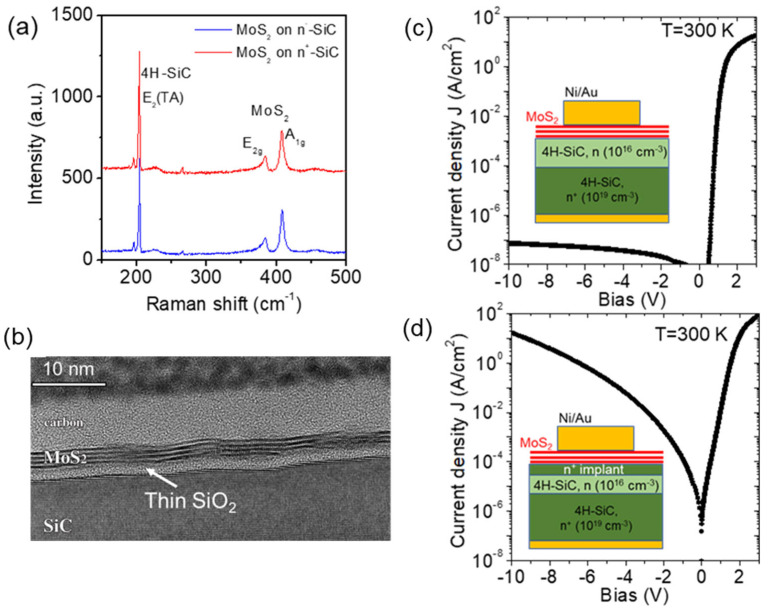
(**a**) Raman spectra of PLD-grown MoS2 on n− and n+-doped 4H-SiC. (**b**) Cross-sectional TEM showing conformal coverage of 3L-MoS2 on the 4°-off 4H-SiC surface, with the presence of an ultra-thin SiO_2_ interfacial layer. (**c**) Current–voltage characteristics, measured at T = 300 K, of MoS_2_ heterojunction diodes with an n− 4H-SiC epilayer and (**d**) n+-implanted 4H-SiC. Image adapted from Ref. [125].

Pulsed layer deposition from an MoS_2_ target under high vacuum is a promising method to deposit high-quality, ultra-thin MoS_2_ films with excellent thickness control on a large area. Recently, this approach has been employed to fabricate n-MoS_2_ heterojunction diodes on 4H-SiC(0001) with different doping levels, i.e., n− epitaxial doping (~10^16^ cm^−3^) and n^+^ ion implantation doping (>10^19^ cm^−3^) [125]. The excellent thickness uniformity (≈3L-MoS_2_) and conformal coverage of the PLD-grown films was shown by Raman mapping (Figure 10a) and transmission electron microscopy (Figure 10b). A wide tunability of the transport properties was shown by the SiC surface doping, with highly rectifying behavior for the MoS_2_/n− SiC junction (Figure 10c) and a strongly enhanced current injection for the MoS_2_/n+ SiC one (Figure 10d). Thermionic emission was found to be the dominant mechanism ruling forward current in MoS_2_/n− SiC diodes, with an effective barrier Φ_B_ = (1.04 ± 0.09) eV and an ideality factor close to unity. On the other hand, the significantly lower effective barrier Φ_B_ = (0.31 ± 0.01) eV and the temperature-dependent ideality factor (n from 4.2 to 3.5 for T from 300 to 400 K) for MoS_2_/n+ SiC junctions was explained by thermionic-field emission through the thin depletion region of n+ doped SiC. The scalability of the MoS_2_ deposition processes and the demonstrated electronic transport tunability on implantation-doped SiC showed great promise for the integration of MoS_2_ with SiC technology.

## 5. SiC MEMS

Micro Electro Mechanical Systems, commonly referred to as MEMS, have existed since the early 1960s, while their first commercialization, at large scale, dates from the early 1980s, with the first pressure sensors. MEMS (or NEMS) are devices of micro- (or nano)-metric size with a mechanical part (generally mobile or vibrating) and an electrical part, allowing the device to function as an actuator and/or a sensor. Most of these MEMS devices are still silicon-based, and have benefited from all advances in microelectronics. However, for various fields, such as aeronautics, space, industrial or even bio and health applications, silicon carbide is the most promising material for MEMS [7,127,128]. Indeed, its outstanding properties (mechanical, physical and chemical), its high level of resistance to temperature, corrosion and radiations, and its biocompatibility largely exceed those of silicon [129]. In this section, we will present the different forms of SiC material used in MEMS technologies and discuss the techniques used to achieve them with a focus on the important process steps. Finally, we will show some past and present device realizations.

### 5.1. SiC Material for MEMS

Although it exists in many different crystallographic forms, the 4H-SiC polytype has become the most common bulk SiC material. It is now available in wafer sizes up to 8″, and is certainly the one used for MEMS technologies requiring bulk wafers [130]. The requested SiC layers for MEMS fabrication, modifying doping and/or thicknesses, are generally obtained by epitaxial growth at high temperatures (>1500 °C). However, there is an extremely interesting crystalline form of SiC for MEMS applications, the 3C-SiC polytype. Indeed, the latter is the only one that can be grown directly on silicon wafers (again, sizes up to 8″). Thus, it benefits from all the advantages of crystalline SiC, but also from all the technological progress made in Si to create MEMS. The 3C-SiC single crystal is obtained by CVD (Chemical Vapor Deposition) growth, either at atmospheric pressure (AP) or, more often, at low pressure (LP) [11,131,132,133,134]. The main constraint is related to the growth temperature, which cannot exceed the Si melting point and which limits growth to approximately 1350 °C. The most-used crystal orientations for the Si wafers (which are generally identical for the 3C-SiC layers) are (100) and (111), although for the latter the thicknesses of the layer remain very limited because of the constraints, which involve cracks [134,135,136]. Many other orientations have been tried [137], but remain anecdotal. A major advantage of these growths is the ability to realize Si/3C-SiC multi-stacks, which will be very useful for MEMS. Other forms of SiC, such as polycrystalline 3C-SiC or (amorphous) a-SiC, are used in these applications either as an active layer or as a protective (coating) layer [138]. The techniques used, apart from those already mentioned, are PECVD (Plasma Enhanced CVD) and ALD (Atomic Layer Deposition) processes [13,14,139,140], generally at low temperatures, which are simpler to implement with, for example, Si electronics associated with the microsystem. A recent review of these materials’ synthesis has been published by M. Fraga et al. [141]. Nevertheless, it is crucial to notice that the mechanical properties of SiC, such as its high Young’s modulus or the layer internal stresses, are in such cases largely affected, and may negatively impact vibrating MEMS structures. Indeed, the Young’s modulus (E) values obtained, through MEMS (clamped beams, membranes) structures, clearly indicate that E strongly depends on a crystalline structure or orientation, thicknesses and doping type and level (by at least a factor of 3, from 120 to 500 GPa) [21,24,142,143,144,145,146,147]. In particular, a strong dependence of the Young’s modulus from both point defects [24] and stacking faults [142] has been reported.

The first attempts reported in the literature concerning the fabrication of SiC MEMS devices are owing to the group of Dr. Mehreghany at the Case Western Reserve University in Cleveland [148], using polycrystalline 3C-SiC grown at relatively low temperatures (typically lower than 1160 °C) with thicknesses in the range of one or few microns. These polycrystalline films could be deposited on many different materials, such as silicon, silicon oxide and polycrystalline silicon, and although their crystalline quality was not outstanding, they showed good mechanical properties, such as a high Young’s modulus and moderately tensile residual stress, which made them suitable for fabricating a variety of MEMS devices [149]. 

Growing SiC on insulating materials such as SiO_2_ is even more convenient than growing it directly on silicon for MEMS fabrication, for electrical isolation issues of the SiC from the underlying substrate. A typical MEMS process that has been used with polycrystalline SiC deposited on SiO_2_ is a surface micromachining process in which the SiC layer is etched through over-selected areas, uncovering the SiO_2_ sacrificial layer that is subsequently removed with an isotropic etching to release the SiC micromechanical structures anchored to the substrate through purposely designed parts that are not completely under-etched in the process. This very simple process permits a variety of devices to be obtained, in which the electrical isolation from the substrate is of fundamental importance, such as lateral and vertical mechanical resonators [150,151]. 

The problem of electrical isolation from the substrate has always been a key issue in SiC MEMS, because most MEMS devices need to have a good electrical isolation to work and, in most cases, the native SiC/Si interface does not provide it. This has always been a problem for using monocrystalline SiC layers in MEMS devices. Such layers certainly provide even better mechanical properties than polycrystalline ones [145,152], but cannot be grown directly on insulating materials such as SiO_2_. Instead, they need to be grown on single-crystal silicon substrates at relatively high temperatures (typically above 1300 °C), to achieve the best crystalline quality and consequently the most outstanding mechanical properties. 

Achieving the required electrical isolation from the substrate with these layers is not easy, since when growing at these temperatures it is not possible to obtain an insulating SiC/Si heterojunction on the epitaxial substrate [153,154]. Consequently, two ways have been explored to achieve this target. The first one is the use of smart-cut technology to transfer a monocrystalline 3C-SiC layer grown from the native Si substrate to another substrate covered with an insulator such as SiO_2_ [155]. With this method, SiC is first grown on a sacrificial Si substrate with the required conditions to obtain a monocrystalline layer and, afterwards, transferred to another oxidized Si substrate using fusion wafer bonding with a subsequent complete etching of the epitaxial substrate (performed, for instance, with KOH or TMAH, but plasma etching is also possible). In this way, a monocrystalline 3C-SiC layer on SiO_2_ is obtained, which can be used to fabricate MEMS devices that can benefit from a perfect electrical isolation from the substrate.

A similar method can also be applied to other SiC polytypes that, differently from 3C-SiC, cannot be grown on silicon (like 4H- or 6H-SiC). In these cases, the same process is used, with the difference that the ion implantation of hydrogen at a controlled depth on the monolithic SiC substrate on which the epitaxial 4H or 6H SiC was grown is utilized to detach the SiC layer after wafer bonding and obtain the desired SiC/SiO_2_ stack for the MEMS fabrication [156]. Using this method, or, in some cases, bulk micromachining techniques from SiC substrates, several MEMS applications have been addressed using 4H- and 6H-SiC, such as pressure sensors [157], thermal sensors [158], accelerometers [159], mechanical resonators [160,161,162], and gyroscopes [163].

The second possible route to obtain monocrystalline SiC layers on insulating substrates (only applicable to 3C-SiC) is trying to grow the layer on SOI substrates, exploiting the isolation provided by the BOX after growing SiC on the device layer. Such a process has been explored by the CNM institute in Barcelona, whose work put into evidence the difficulties related to the high temperature needed to grow high-quality monocrystalline 3C-SiC layers and the related damages that can take place in the BOX because of them, with possible negative consequences on electrical isolation [164]. However, the successful fabrication of etching-isolated electrostatic resonators on the SOI substrate has been reported more recently [165], despite the high growth temperature of the SiC layers, starting from very low doping SOI substrates that seem to prevent the damage of the BOX, possibly related to the diffusion of the doping in it during the high temperature growth of SiC.

### 5.2. SiC Micromachining Technologies and Processes 

For MEMS manufacturing, two major techniques are generally used: bulk and surface micromachining. Bulk micromachining (BMM) generally consists of machining the volume of the wafer in order to define the future vibrating structures [144]. However, when bulk is purely SiC, its very high chemical inertness makes this operation extremely delicate. If, for silicon technologies, chemical etching is used, it is almost inoperative in the silicon carbide case except when using very high process temperatures (>300 °C). Several ways remain possible for this technology. Thus, electrochemical etching processes, mechanical polishing, or laser ablation techniques have been used [144,166,167,168]. Plasma etching is, of course, possible but, for bulk SiC, time and cost are prohibitive on an industrial scale [168]. An alternative is to use a Si wafer, which is itself machined, and on which a layer of SiC is deposited (before or after) [169]. In such cases, usually only polycrystalline or amorphous SiC is possible. Furthermore, wafer bonding can also be a solution for both technologies (BMM and SMM) [7]. Surface micromachining (SMM) is probably the most used technique for SiC-based MEMS [7,127]. For SMM, Si substrates are mainly used, allowing them to benefit from all the advances made in Si technologies. SMM generally consist of multilayer stacks, where some of the layers are used as a sacrificial layer and are etched in particular areas to form the structures. When one wishes to take full advantage of the exceptional mechanical properties of SiC (such as a high Young’s modulus, allowing an increase in the vibration frequencies of the systems compared to Si with an identical design) or to add electronic functions close to the MEMS, single crystal 3C-SiC is the material of choice [145,170,171,172]. Furthermore, it has been shown that it is possible to grow multi-layers of 3C-SiC/Si monocrystalline [173,174]. In cases where only chemical inertness and/or radiation resistance properties are of interest, polycrystalline 3C-SiC and a-SiC are the most commonly used materials, as they are the easiest forms to obtain. The classically used layers in microelectronics, such as Si, Si-poly, SiNx, SiOx or metals, can be easily etched, by wet as well as by dry processes, and this, in a selective way, is not the case for the SiC layer. The etching of the SiC layer (mono- or poly-crystalline, as well as amorphous) is essentially carried out by the plasma technique, whether for the classical RIE (Reactive Ion Etching), RIE-ICP (Inductively Coupled Plasma) or even, but more rarely, IBE (Ion Beam Etching). The chemistry used is generally fluorine chemistry based on SiFx and CFy [175]. Many masks for RIE technologies have been tested as thick photoresists, or SiOx and Al_2_O_3_ without convincing success. The most efficient mask seems to be the hard mask using Ni, which is related to its inertness to the species present in the plasmas used [21]. The RIE or ICP etching processes of SiC are nowadays still far from the performances that are possible on silicon (such as those provided by cryogenic or DRIE etching), both in terms of maximum depth and anisotropy. Generally speaking, it is relatively difficult with these processes to obtain etching features with a very high aspect ratio, because of problems related to the resistance of the masks to the etching processes and inherent performance limitations of the plasma processes. That is the reason why improving these SiC etching processes in MEMS fabrication is still, today, a very important research target for many groups [163,176,177,178,179,180,181]. 

**Figure 11:**
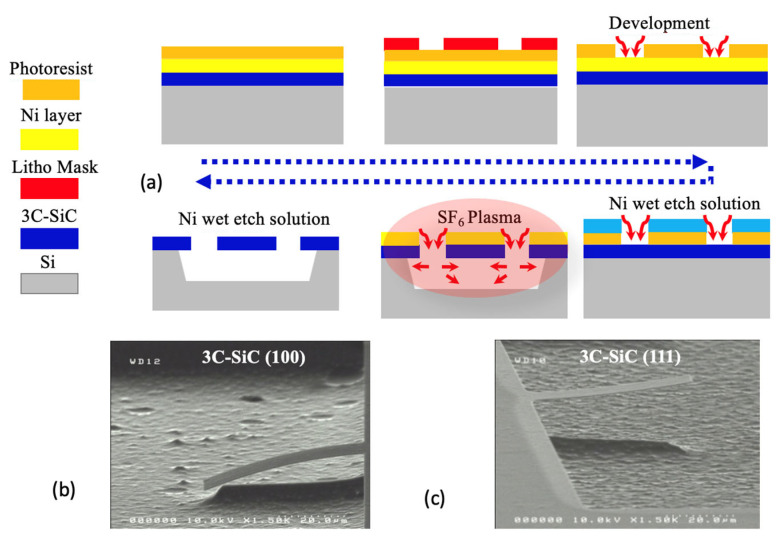
(**a**) Cross-sectional schematics of the 3C-SiC micromachining process and SEM images of clamped beams obtained by this process dependent on orientations (**b**) 3C-SiC (100) and (**c**) 3C-SiC(111) bending, respectively, downward and upward (issue from [143]).

In order to illustrate a simple micromachining process, Figure 11 describes the realization process of clamped beams and shows, in a real case, beams obtained for 3C-SiC (100) and (111) (work related to [21,143]). Furthermore, one can observe the impact of the stress on these structures, which is related to the growth process and the orientations of the original Si material, as mentioned in the previous paragraph.

### 5.3. SiC MEMS Devices

**Figure 12:**
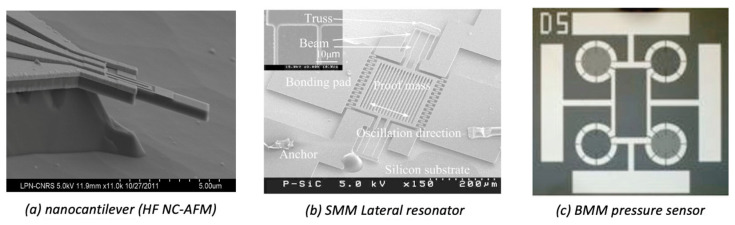
Examples of 3C-SiC devices taken from the literature: (**a**) a nanocantilever for High frequency Non-Contact AFM (from [182]); (**b**) a lateral resonator obtained by SMM (from [183]); and (**c**) a pressure sensor with Si BMM process (from [169]).

In recent years, many advances have been made in SiC technologies and have benefitted MEMS made of SiC-based materials. The diversity and the number of available devices has greatly increased and can be clearly seen in the bibliography/reviews presented here. The targeted application fields are also much wider today. If the domains where one meets severe environments, such as aeronautics, space and energies (fossil and nuclear), remain the most frequently targeted ones, others, such as biology or instrumentation, clearly still appear. It would be illusory to present an exhaustive list of the MEMS types already realized of functional SiC-based MEMS devices by an increasing number of research groups (both for academia and industry). One can now easily find many pressure and gas sensors of all types, accelerometers, resonators, micromotors, cantilevers, tips, etc. In order to illustrate the diversity of devices, we present in Figure 12 two examples of MEMS obtained by SMM, and one by BMM. Finally, there is a last problem that has not been addressed in this section, but which is extremely complex: the packaging of MEMS structures. Indeed, it is very delicate, and even more so in view of the targeted applications, to encapsulate, protect and connect these objects to the outside world. This problem, often forgotten by the teams working on MEMS devices, certainly deserves to be further developed.

To conclude, although they are still complex to realize and progress on materials, as well as on micromachining processes, is still necessary, MEMS are widely open to silicon carbide. The SiC allows, indeed, for the microsystem’s exceptional chemical and physical properties to exceed the classical limits in which MEMS were confined until now.

## 6. SiC Photonic Devices

SiC is emerging as an interesting material platform for PIC and QPIC. A couple of comprehensive review papers have been published recently, addressing its advantages in nano photonics, quantum photonics and spintronics. This section will only cover very general concepts in this field.

To explore the unique optical properties of SiC, a structure named optical waveguide is indispensable. In a SiC waveguide, the light is confined in the SiC core, which is surrounded by low refractive index material such as air, silicon dioxide (SiO_2_), etc. To achieve the tight confinement of light in the SiC core, SiC waveguides normally have a cross-sectional geometry of sub-microns. Therefore, the biggest challenge for SiC optical device development lies in a so-called silicon carbide on insulator (SiCOI) stacks formation, because the commercially available 3C SiC epilayer grown on silicon substrate and SiC wafers are not ready for the optical devices’ fabrication. The exploration of 3C SiC optical devices was initiated by etching the Si near the 3C SiC waveguide to form the index contrast. However, optical devices formed in this way lack mechanical stability and restrain the further fabrication process to enhance the functions of devices such as applying contacts for active devices. Therefore, different film transfer technologies have been developed to form both 3C and 4H SiCOI stacks. For 3C SiCOI, a 3C SiC layer is bonded to a low refractive index substrate and then the Si substrate is removed. After polishing the SiC surface, it is ready for optical device fabrication. For 4H SiCOI, the starting material is 4H SiC wafers. Therefore, after bonding, two different technologies are developed to thin down the SiC layer: ion cut and grinding. Ion cut (also called smart cut) is a method adapted from silicon on insulator (SOI) formation. The grinding method reduces the thickness of SiC from around 350 to 500 µm down to 1µm, which is very expensive because most of the SiC material is wasted. Right now, 4H SiCOI outperforms 3C SiCOI because of the better crystal quality of 4H SiC. In comparison to the ion cut method, the grinding method delivers a better performance of 4H SiCOI because the defects generated during ion implantation could not be recovered completely after post annealing, even at 1300 degrees C limited by the Si substrate. Therefore, 4H SiCOI formed by the grinding method plays a dominant role in high-performance SiC photonic devices.

When a SiCOI stack is ready, a waveguide device is fabricated normally through three main steps: nanopatterning resistance by electron-beam lithography or deep ultraviolet stepper; transferring the pattern from resistance to SiC through reactive ion etching (RIE); and depositing the SiO_2_ layer on top of the SiC to protect it from environmental disruption through plasma-enhanced chemical vapor deposition (PECVD). It is clearly seen here that the fabrication of SiC optical devices harvests the already-existing CMOS process. Low propagation loss is a fundamental requirement, which could be achieved by minimizing the material loss and fabrication-induced loss (SiCOI formation and etching). Although SiC waveguide devices are a new research area, the propagation loss is already as low as the other main material platforms, such as Si.

For PIC applications, point defects are generally not desirable because they act as absorption or scattering schemes, which lead to a high loss of the device. Right now, the best results (lowest loss) achieved for SiCOI waveguides are those using high-purity 4H SiC and formed with the direct bonding and thinning down process, which explains the importance of having a low density of point defects compared to the higher loss caused by the higher density of point defects in 3C SiC and 4H SiCOI formed by the smart cut method. However, versatile point defects in SiC also play a constructive and active role in QPIC because they could potentially be single photon emitters working at room temperature, and their emission wavelengths cover from near UV to telecom. In this case, a single point defect is placed precisely at a desired place, such as a microcavity, so it has only a very minor effect on the other optical devices. In other words, the induced loss by the point defects could be ignored.

**Figure 13:**
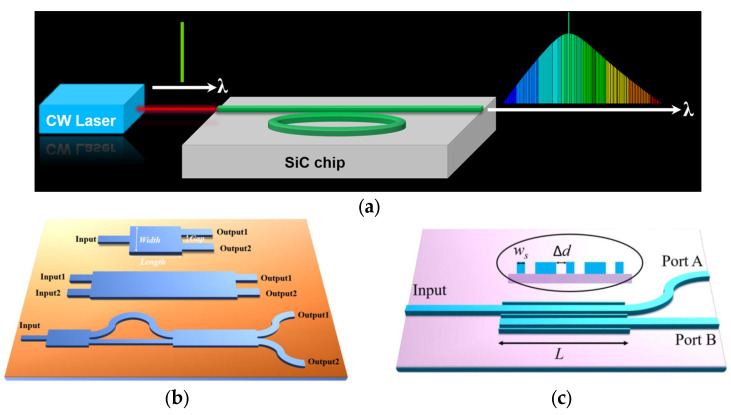
Schematic of basic building blocks to construct optical chips: (**a**) An optical frequency comb light source; (**b**) 1 × 2 multimode interference (MMI), 2 × 2 MMI, and Mach-Zehnder interferometer (MZI) splitter; and (**c**) polarization beam splitter with vertical-dual-slot waveguides.

Depending on the design of the patterns, the light confined in the waveguide could be manipulated through coupling, phase control, interference, wavelength conversion, absorption, etc., therefore demonstrating the different building blocks (light source, polarization splitter, optical switch, optical memory, optical isolator, photon detector, etc.) of an optical chip. Figure 13 shows three building blocks as examples.

**Figure 14:**
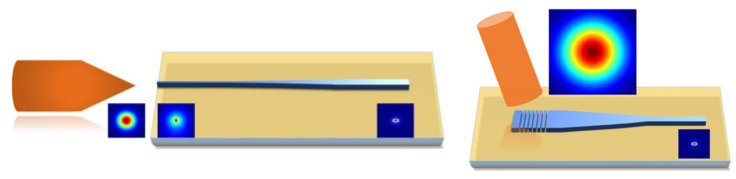
Schematic of two different coupling schemes: (**a**) edge coupler and (**b**) grating coupler.

Additionally, an efficient coupling scheme is crucial for an optical chip. There are usually two types of coupling schemes: butt coupling and vertical coupling, shown in Figure 14. For butt coupling, a standard single mode fiber approaches the edge of the chip where the end of the waveguide is located and aligned. This scheme requires the cleaving of the chip to expose the end of the waveguide. In case of the mismatch of mode profiles supported in fibers and the waveguides, a taper fiber and an on-chipmode converter are implemented to minimize the coupling loss. Advantages of this scheme include a wide working wavelength range and polarization independence. For vertical coupling, as shown in Figure 14b, a grating coupler is implemented where a standard single mode fiber vertically approaches the grating coupler, which could be placed anywhere on an optical chip, eradicating the risk of killing the chip during the cleaving process. Although a vertical coupling scheme facilitates the on-chip test flexibility, it has a limited working wavelength range and is polarization-dependent. 

The reason for co-existing multiple material platforms for PIC is that no material platform has ideal properties to realize all the basic building blocks while, on the other hand, also providing a good opportunity for SiC, a newcomer in the PIC field. The past decade has witnessed rapid progress in SiC-integrated photonics.

First of all, 4H SiCOI stacks have been developed with 95% yield and up to 4″ wafers [184].

Then, a lot of building blocks have been demonstrated with good performance: highly confined SiC waveguides with an efficient coupling scheme [185], beam splitter [186], polarization beam splitter [187], optical parametric oscillation [188], optical frequency comb [189] and optical modulator [190].

The yet-to-be-demonstrated basic building blocks direct the most interesting research directions for SiC in future. Non-reciprocal building blocks, such as the optical isolator, remain a big challenge for the main material platform of Si. Therefore, exploring the unique optical properties of SiC to solve such a challenge is a good arena in which SiC can play and win.

The final goal is to integrate these basic building blocks to realize powerful functionalities on one chip, which will provide a solution to the challenges faced in integrated circuits.

## 7. Biological Performance of SiC

In this section, we discuss the materials-level interaction of SiC and present some compelling work that shows that not only does SiC display excellent levels of compatibility with biological tissue, but that it may be functionalized to further enhance its interaction, which is key to realizing in vivo biosensors. 

Cell–semiconductor hybrid systems are an important component of many biotechnological applications [190]. One in vitro biocompatibility study of the main three SiC polytypes was conducted by Coletti et al. [191]. The reported results document the in vitro biocompatibility of all principals’ SiC polytypes, and their capability for directly interfacing cells without the need of specific surface functionalization. Numerous cell lines were studied to better understand competing literature reports that suggested that SiC was both bio-permissive and cytotoxic. By studying the main crystal forms of SiC, the apparent contradiction turned out to be likely due to nuances in how SiC should be prepared for cell culture. In the final analysis, SiC, and its numerous polytypes and forms (amorphous, polycrystalline, monocrystalline, nano-structured, etc.), were all shown to provide an excellent interface to the biological world [30]. 

One of the important properties of implantable biodevices is hemocompatibility. This is particularly true for neural implants, due to the dense vasculature in the brain. Two studies were conducted to assess hemocompatibility, namely the interaction of platelet-rich plasma (PRP) with various SiC and control surfaces in both static [31] and dynamic [30] modes. In the static study, it was observed that 3C-SiC produced little thrombotic reactivity, especially compared to Si when contacted with blood platelets, which was not surprising as Si is known to stimulate the clotting cascade (i.e., thrombosis). Interestingly, the hexagonal polytypes tested (4H- and 6H-SiC) were no better than Si when in contact with PRP [31]. Amorphous silicon carbide, a-SiC, an insulating material that can be used for biomedical devices, was not tested in the initial experiments. Consequently, Nezifati then examined dynamic thrombotic conditions using a flowing Chandler loop to mimic the cardiovascular system [30]. A histogram comparing platelet adhesion to various surfaces clearly demonstrated the low platelet activity of 3C-SiC, especially compared to Si and SiO_2_. Interestingly, a-SiC only displayed a slightly lower platelet reaction when compared to Si under dynamic conditions.

**Figure 15:**
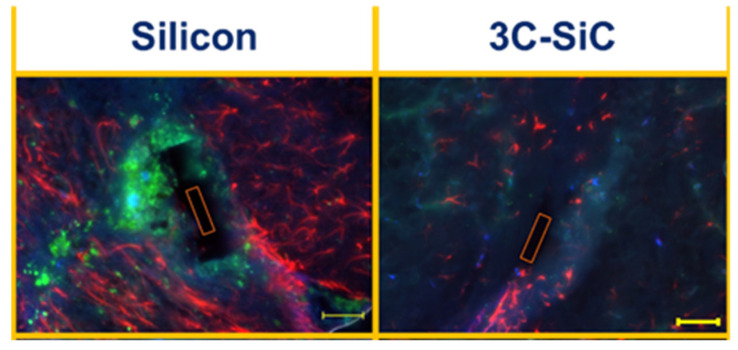
Digital photographs from immunofluorescence analysis of C57BL/6J mouse hippocampal tissue. Left shows tissue response within the left hemisphere of the hippocampus surrounding the Si implant, and right presents tissue of 3C-SiC within the right hemisphere. Scale bars are 50 µm in length, images from [29]. Red rectangles mark implant dimensions for reference. Green = CD45 dye indicates microglia/microphages, Red = GFAP indicates Astrocytes, and Blue = MAP2 indicates neuron dendrite cell body. No inflammatory response detected for 3C-SiC implant, while Si shows a significant response and neural tissue loss.

Several in vivo studies were performed at the University of South Florida, which served as further motivation for the development of SiC biomedical devices. Three (3) animal models were studied (mouse, rat and pig) and, in each case, a null immune system response to 3C-SiC and a-SiC was observed, whereas a significant stimulation of the immune system was observed for Si. The first involved non-functional double-shank probes, with Si on one side and 3C-SiC on the opposite, were implanted into C57BL/6J wild-type mice for 35 days. A comprehensive histological analysis of 3C-SiC for neural device applications was performed and reported for the first time in [30]. Perhaps the most important outcome of this work was the in vivo tissue comparison between identical form-fit Si and 3C-SiC passive double-shank devices. After harvesting tissue slices, the Si and 3C-SiC implant sites were compared on the same slice from three (3) different mice, with the resulting tissue histology data displayed in Figure 15.

Several important observations can be made from the images related to the implant location. First, the left and right images are from the same tissue slice and show the areas where the Si and 3C-SiC implants were located, thus allowing for a meaningful direct comparison between the two materials within the same animal. A large area devoid of neural tissue is apparent around all the Si implants. Additionally, the voids are often larger than 50 µm wide, which is a distance much larger than the implant thickness of ~20 µm. The voids for the areas where the 3C-SiC implant was removed are much smaller in comparison, and closer to the original size of the implant, which suggested that less overall damage has occurred to the brain. While GFAP^+^ and CD45^+^ activity are increased in the areas of the implants for Si, 3C-SiC shows little appreciable activation. Based on this work, functional SiC neural implants have been developed and are presented later in this paper and in [29].

These studies suggest that fully monolithic “All-SiC” biomedical devices, i.e., devices composed completely of SiC materials, may be homogeneously integrated for long-term human use, having clear advantages over Si- or metal-based devices. They also suggest that SiC may be beneficial for other biomedical devices, as well. In many neuroscience applications, long-term implantation is not a major issue and simply coating contemporary neural interfaces with *a*-SiC insulation provides numerous benefits. The work carried out by Pancrazio et al. has shown a significant in vivo improvement of SiC-coated implants, as reported in [192].

### 7.1. SiC Biosensors

Numerous approaches to biosensing are available in the literature, and it is simply too exhaustive a list to provide here. An overview of SiC biosensing that encompasses what is provided in this section may be found in [193]. Common to many biosensors is the need to functionalize the sensor surface to achieve adequate specificity (i.e., discrimination between target analytes and other components in the biosensor environment). In [2], some examples of self-assembled monolayer (SAM) formation with organosilanes and short chain alkenes, as well as direct surface photopolymerization using vinyl monomers, were reported. In fact, a case example of how SAM-modified surfaces can be used to control the spatial wettability of SiC for biomedical applications was provided in [2]. The SAM-based bio-functionalization of 6H-SiC was performed to assess its impact on cell morphology and substrate permissiveness. The SAMs were formed by reaction with aminopropyldiethoxymethylsilane (APDEMS) and aminopropyltriethoxysilane (APTES), using the silanization techniques described in [2] to create moderately hydrophilic surfaces. The quantification of cell proliferation was achieved using MTT assays, which were performed in accordance with [191]. 

SiC has long been used in advanced radio frequency systems due to its excellent carrier transport and high-power properties. Many of today’s point-of-use healthcare systems are hybrid implantable systems that combine radio frequency (WiFi and/or bluetooth) and biosensor technologies. Biosensors usually rely on relevant physiological parameters for continuous monitoring, and an integrated antenna is often employed to send the received data to an external receiver. Hybrid systems combining RF (antenna/wireless communication) and biosensor technologies are key to developing the next generation of continuous monitoring systems, including implantable pacemakers and defibrillators, glucose monitors, insulin pumps, hearing aids, health care facility communication, medical and emergency equipment tracking and remote patient monitoring, just to name a few.

A continuous glucose monitoring (CGM) sensor employing radio frequency (RF) signals was demonstrated using 4H-SiC [30]. The fabricated implantable RF antenna utilized semi-insulating SiC as the material of choice due to its great robust bio- and hemocompatible material for long-term in vivo systems. Distinguished by its innovation to utilize SiC as the device material, this system removes the need to encase this biomedical device inside biocompatible materials, thus addressing the short lifetime issue experienced by many contemporary sensors. 

Unlike biosensors, that require direct contact with interstitial fluids to trigger chemical reactions with functionalized surfaces, this SiC sensor does not require a direct interface with bodily fluids. The sensing mechanism is based upon shifts in the antenna resonant frequency as a function of changes in glucose levels electrically manifesting as corresponding shifts in blood permittivity and conductivity.

From these results, it was observed that the antenna responses to both a blood mimicking liquid and actual pig blood had similar frequency shift trends, where the resonance frequency decreased with an increase in glucose levels. It was noted that the SiC-based sensor covered the human normal to critical care glucose regions with a reasonable slope in the frequency vs. glucose response at 10 GHz. These results demonstrate the potential use of biocompatible SiC to fabricate the long term, real-time continuous glucose monitoring of diabetic patients. The sensor was then re-designed to operate in the industrial, scientific and medical (ISM) (5.8 GHz) band, and this work, while still on-going, has been quite promising. This non-invasive solution would be much more convenient for the patient and would not require surgery, and lower power WiFi communication would be allowed due to there being no signal attenuation in human tissue.

### 7.2. SiC Nanotechnology

Nanotechnology is a very broad field of exploration and technical development, which means different things to different people. Most of the reported works involving SiC nanotechnology use either nanoparticles or nanowires. For a more general discussion of SiC nanotechnology, involving other applications, an excellent review article is available [194].

Nanotechnology enables innovative systems with unique properties and applications in several fields, from sensors to nanomedicine [195]. For health care applications, the ability to tailor the material properties allows for the design of new nanosystems with enhanced performance for diagnostics, imaging and oncotherapy [195,196]. Nanowires (NWs) based on 3C-SiC have a strong potential since they are chemically inert and compatible in the biological environment. Indeed, 3C-SiC has proved to be a bio- and hemo-compatible material, and some biomedical device prototypes have been successfully realized through thin film SiC fabrication, as reviewed in [29]. The combination of 3C-SiC with silicon dioxide in core/shell NW structures (i.e., core/shell 3C-SiC/SiO_2_ NWs) opens more ways to engineer the surface via functionalization and decoration with macro-molecules and nanoparticles [128,197]. The potential use of core/shell NWs in nanomedicine is driven by the presence of the amorphous shell, since it can modify the material behavior in the biological environment, while for blood-contacting applications, SiO2 typically induces an aggregation and activation of platelets, promoting clot formation and acute inflammatory processes [15,198]; evidence from the 3C-SiC core/SiO_2_ shell NW system has not shown a deleterious impact on cells in vitro [199]. Further, a peculiar feature of the core/shell NWs is their optical emission: the oxide shell enhances the core luminescence [199,200,201] when the nanosystem is excited by highly energetic sources, such as electron beams or X-rays. This property opens the possibility of exploiting 3C-SiC/SiOx NWs as radiation-resistant scintillation nanostructures, which can be properly functionalized to play an active role for new oncotherapies; for example, X-ray excited photodynamic therapy (PDT) [201] in nanomedicine.

While point and crystal defects continue to be an issue for SiC power device applications, for SiC neural probes they appear not to be a serious issue due to the relatively low voltages involved during bi-directional signal transduction: neural signals are typically on the order of 10–100 mV when recording from the brain, and stimulation involves a maximum voltage amplitude of 5 volts, with typical stimulation waveforms in the 1–2 Volt range. Indeed, interdigitated electrode (IDE) measurements on both 4H- and 3C-SiC showed negligible leakage currents within this stimulation range, while the 4H-SiC IDE devices demonstrated leakage currents of less than 1 nA to ±50 V, even in a liquid PBS solution. Therefore, defects in the SiC mesa electrodes appear to be of negligible concern for this application, as discussed in further detail in Section 5 of [29].

The work by Salviati et al. [31] provides an overview of the growth and optical properties, as well as some in vitro applications, of core/shell 3C-SiC/SiO_2_ NWs and bare SiC NWs. It was shown, by the analysis of cell proliferation, cell cycle progression, and oxidative stress, that the core/shell NWs are cytocompatible over a time of up to 10 days. They are effectively internalized by cells through the macropinocytosis mechanism (phagocytosis only in the THP-1 model), and sporadically by direct penetration. For all the cell lines studied, the intracellular presence of NWs induced the same molecular events: the peroxidation of membrane lipids, and the oxidation of proteins. These effects are late stage, and may be limited by the activation of protection systems; for instance, the effect of ROS is not acute and effectively countered by the intracellular scavenger. These results highlight that core/shell 3C-SiC/SiO_2_ NWs do not elicit either midterm (72 h) or long-term (10 days) cytotoxic activity. 

While singlet oxygen generation does kill cancer cells, it also kills healthy cells within close proximity. An alternate approach was proposed and studied which sought to use SiC nanostructures to facilitate cancer treatment via photo immune therapy (PIT). The concept is to enable ‘deep-tissue’ cancer treatment by using X-ray irradiation to stimulate 690 nm NIR (near IR) emission from SiC nanostructures to activate the PIT process using antibody conjugates containing the IR-700 molecule. This research was specifically aimed at treating patients with such pernicious cancers as liver, pancreas, etc. So far, a very promising SiC-based core–shell nanostructure system (ZGC:SiC) has been developed and reported by Beke et al. from this project [202].

Semiconducting nanowires are of a growing interest for nanoelectronic devices, for sensing applications in particular [9]. While silicon is the most widely used material in this field, it lacks long-term stability in aqueous solutions [203,204] and, therefore, is not suited for most liquid environments. Indeed, chemical resistance to the medium is essential for device reliability and signal stability. SiC appears as a promising material for these applications, thanks to its semiconducting properties as well as its very high chemical stability and biocompatibility [29]. However, SiC is much more difficult to etch due to its high physical and chemical stability, compared to Si material. Thus, core–shell Si/SiC nanowires could be an advantageous compromise between these two approaches [205]. Applications include ion-sensitive FETs (ISFETs), chemical FETs (ChemFETs), and other related sensing devices. These devices would benefit from the strong chemical and physical stability of the SiC shell and from the superior electron transport within the Si core. An excellent recent work may be found in the literature by Zekentes et al., in which numerous SiC nanowire sensor applications are presented, including FET biosensors and applications [15].

### 7.3. SiC Implants

Implants refer to the general class of devices/materials that are surgically inserted into the human body. They typically involve dental prostheses such as artificial teeth, cardiovascular stents to prevent vascular collapse after angioplasty or biosensors such as those used in modern glucose monitoring systems. There are also skeletal (bone) implants, traditionally made with metals, for such common applications as artificial knees and the like. In the case of bone implants, they are often treated to display a ceramic surface to tissue to reduce and/or eliminate irritation and thus reduce body rejection. Perhaps the most interesting application of SiC implants involves the human nervous system, either central (brain) or peripheral (limbs). In this section, we will review some excellent examples of the use of SiC both in bone and to provide a bi-directional signal pathway to the nervous system. 

### 7.4. Bone Scaffolds

Polycrystalline silicon carbide (SiC) has been extensively used in the ceramics industry and is mainly known for its mechanical strength and refractory characteristics. Hence, it is used extensively as a grinding agent as well as a high-strength machine tool coating. As examined previously, SiC is highly biocompatible and, when implanted, has demonstrated minimal inflammatory or null tissue response [30]. In an effort to explore the potential of SiC as a substrate for bone growth, nano porous SiC (np-SiC) was mineralized with sol-gel coatings of hydroxyapatite (HA), under various deposition conditions, as described in [29]. Osteoblasts were found to proliferate at higher rates than HA-coated Si, thus indicating that bone scaffolds made from np-SiC may prove advantageous for many applications where bone transplants/implants are required.

Dental implants are perhaps one of the most common devices implanted in the human body for long-term use. There are several issues, mostly relating to the osseointegration and biocompatibility of the implant to tissue (soft and bone). Some examples of superior implants using SiC-coated dental implants are reported in [198]. One of the important advantages of using SiC for dental implants is the natural bio-fouling resistance of this material, which is discussed in detail by Franca et al. in [29]. Biofilms are inhibited when SiC surfaces are presented to biological matter [29]. This property, along with the structural properties of SiC, makes this an ideal material for dental implant applications.

The potential impact of permanent neuro-compatible implantable devices to assist millions who have experienced brain and spinal cord injury and/or limb loss is tremendous, both in restoring patient functionality as well as in quality of life. Until now, no known reliable solution to this challenge has been found, with most of the current technology relying on materials not compatible with the neural environment, such as inorganic materials including silicon, tungsten or platinum, or polymer-based insulators such as parylene-C and polyimide. SiC, and in particular 3C-SiC, appears to offer material properties which would meet this challenging application: the evidence of bio- and hemocompatibility, tailorable doping profiles for the seamless integration of electronics, high material durability within harsh, corrosive environments, and lastly, excellent thermal conductivity [29]. 

The only neural implant approved for limited human use to date is based on the University of Utah intracortical array [7]. Due to numerous challenges, long-term reliable performance has yet to be achieved, some of which has been attributed to heterogeneous material fabrication consisting of the silicon shank, platinum/iridium-based electrode conductors, and parylene-C insulation.

**Figure 16:**
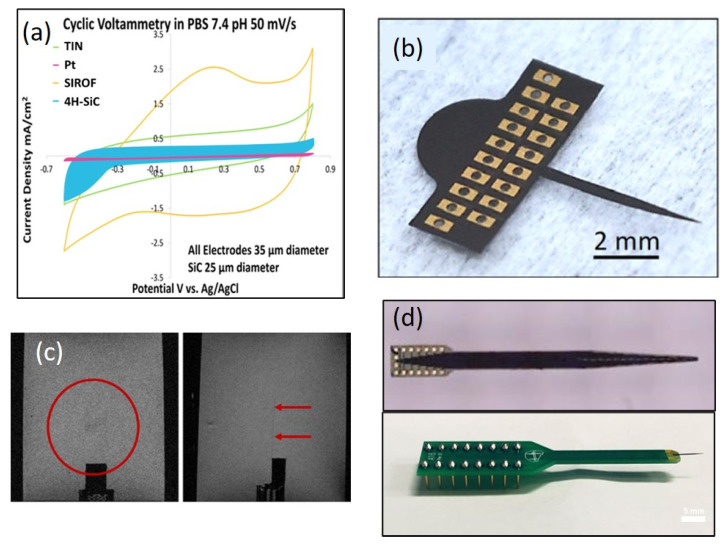
Examples of SiC INI devices developed in the USF SiC Group. (**a**) CV performance comparison of an all-SiC (4H-SiC polytype) MEA, showing superior performance to Pt electrodes of the same form-fit. (**b**) Free-standing all-SiC (3C-SiC on SOI) 16-channel functional INI used to assess the MRI compatibility. (**c**) Sagittal (left) and coronal (right) MRI images @7T showing no image artifacts, and nearly transparent performance of 3C-SiC in a brain-tissue phantom. (**d**) Photographs of a released C-based *a*-SiC INI device ~1 µm thick (top) and a packaged device (bottom) with Si backing for structural support mounted on a Neuro Nexus package header for in vivo testing [29].

Fortunately, numerous instances of SiC being used as a robust material for implantable neural implant (INI) applications have been reported [9,201,202,203,204]. The USF SiC group has been working on this challenge for nearly two decades, and have developed several possible solutions to address long-term in vivo INI issues: an ‘all-SiC’ monolithic MEA constructed using 4H-SiC [128], an alternate device based on 3C-SiC on either bulk Si or SOI substrates [9] and, most recently, an ultra-thin interface using *a*-SiC as the base and capping insulation, which sandwiches a carbon electrode created using pyrolyzed photoresist film (PPF) [204]. Not only has excellent electrical performance been demonstrated in PBS, but in the case of the 3C-SiC ‘all-SiC’ interface on SOI, excellent MRI compatibility has been observed in a 7T animal bore MRI system at the Moffitt Cancer Center [203]. Details of this research may be found in [29]. A preview of these results is shown in Figure 16.

A comparison of both the all-SiC and C-based SiC electrodes has been complied by C. Feng and is shown in [204].

## 8. Conclusions and Outlooks

In this review, we have reported several new applications of SiC that can have a large impact on several fields (electronics for harsh environment, radiation hard detectors, high frequency MEMS, optical chips, in vivo devices). The development of these new devices has been trained from the continuous improvement of SiC technology and of the material quality and price, due to the increasing market for power devices. However, at the same time, these applications need some specific features that can be also of great interest for the power devices.

Reliable high-temperature (>400 °C) SiC electronics will someday offer substantial improvements to aeronautics and space vehicles, especially when sensors, integrated circuits, and high-power devices can all be combined to form compact integrated subsystems free of environmental sheltering requirements. However, significant further technological advancement, of both SiC as well as integrated packaging, towards achieving reliable operations for such extreme aerospace environments remains to be accomplished before this vision can become realized. 

SiC complimentary devices such as CMOS and CJFET are attractive for low-loss ICs operating under harsh environments owing to their complimentary configuration. Various SiC CMOS ICs have been successfully demonstrated, though the mobility improvement and threshold voltage instability, especially for p-channel MOSFETs, are still challenges. SiC CMOS devices can also be used for the gate driver and sensing circuits of a high-voltage SiC power MOSFET on the same chip, leading to high-voltage power ICs, which outperforms Si devices. On the other hand, SiC CJFET ICs are free from the oxide-related problems, but are still in the early stage. Major challenges with SiC CJFET include the threshold voltage control and the improvement of the mutual conductance.

The use of silicon carbide for the construction of radiation detectors looks very promising. An extremely low leakage current and resistance to radiation damage are definitely the main advantages. Certainly, in the near future, we will see ever-more-sophisticated and performing devices designed to be used for the detection of radiation (charged particles, neutrons and photons) in scientific studies or in applications in the field of health, environment, energy, cultural heritage, etc.

We have shown that the integration of 2D materials such as epitaxial graphene and MoS_2_ on 4H-SiC provides additional functionalities to the SiC substrate, enabling advanced or new applications beyond power devices (e.g., RF transistors, quantum Hall resistance standards and environmental, chemical and biological sensors). Furthermore, the possibility of tuning the vertical current injection at the 2D materials/SiC interface, either by the intercalation of properly chosen atomic species, or by doping 2D materials and/or 4H-SiC, can be exploited for engineering vertical heterojunction diodes for power electronics.

In the case of SiC photonic devices, this is still in its early phase of research, benefitting from the advances of nanofabrication and SiC material growth technology. The past decade has witnessed ground-breaking results, starting from scratch in SiC-based nonlinear photonics and quantum photonics. It is paving its way in very competitive multi-material platform PIC and QPIC fields, thanks to its unique optical properties. Looking forward, the co-integration of SiC electronic and photonic devices to address the market’s needs could attract more investment for both research and commercialization, which will hopefully accelerate the release of the full potential of SiC in photonics in the near future. 

Due to its phenomenal physical properties, SiC is the ideal material candidate for MEMS devices in harsh environments and severe operating conditions. Even if the realization remains complex, the tremendous progress on materials, available now up to 8” and with a high crystalline quality, on deposition techniques, as well as on the micromachining of the layer (active or sacrificial), means that SiC MEMS prototypes for many applications are now widely available. Despite these accomplishments, few devices have succeeded in reaching commercialization. Indeed, efforts towards technological milestones, interfacing with full SiC electronics as well as the availability of capacity in the SiC foundry, are still required if we do not want to confine SiC MEMS to demonstrators or small, niche markets. The high potential of SiC for MEMS for the future of transportation, space, industrial, bio and health applications is real and must be developed.

In this paper, a review of SiC as a robust and highly effective material for biomedical devices and applications has been presented. Consequently, the future of SiC as a material for the design of advanced biomedical devices appears to be very bright. While market analysis has yet to be undertaken, the global medical device market size was USD 432.23 billion in 2020 and is projected to grow to USD 657.98 billion in 2028 at a CAGR of 5.4% in the 2021–2028 period [206]. One can speculate that SiC biomedical technology will play an important role in this global market, as the long-term reliability of this class of in vivo medical devices is simply unparalleled. The hope is that this paper will motivate biomedical technologists to consider the development and adoption of SiC for next-generation medical devices.

## 9. Patents

The BioSiC research reported here has resulted in several patents over the years. As the work included here is a review of prior publications, please refer to those for further information.

## Figures and Tables

**Table 1:** Main properties of 3C-SiC, 4H-SiC and Si.

Property	3C-SiC	4H-SiC	Si
E_g_ [eV]	2.4	3.28	1.12
E_breakdown_ [V/cm] @ 1 × 10^16^ cm^−3^	1.5 × 10^6^	2.5 × 10^6^	3 × 10^5^
μ_e_ [cm^2^/Vs]	900	1000–1200	1450
μ_h_ [cm^2^/Vs]	40	115	450
V_sat_ [cm/s]	2 × 10^7^	2 × 10^7^	0.8 × 10^7^
Z	14/6	14/6	14
ε_r_	9.7	9.7	11.9
e-h energy [eV]	-	7.6–8.4	3.6
Density [g/cm^3^]	3.22	3.22	2.33
Displacement [eV]	-	30–40	13–15
Young’s Modulus (GPa)	450	390–690	160
Hardness (GPa)	35–45	21	7
Poisson Ratio	0.18	0.21	0.22
Thermal conductivity [W cm^−1^K^−1^]	3.2	3.7	1.5
Refractive index @1550 nm	2.6	2.6	3.48
Nonlinear refractive index [m^2^/W]	5.31 × 10^−19^	8.6 × 10^−19^	6.7 × 10^−18^
Second-order susceptibility [pm/V]	33	0.3–0.7	0

**Table 2:** Advantages and concerns of SiC CMOS and CJFET devices.

Device	Advantages	Concerns
SiC CMOS	High input resistance; Flexible supply voltage (2–20 V); Large benefits from Si CMOS.	Low channel mobility; Threshold voltage instability; Oxide reliability in harsh environment.
SiC CJFET	Free from oxide reliability issues; Superior resistance against high temperature and radiation; Easy SPICE simulation.	Severe threshold voltage control; Limited supply voltage range (≤2.5 V); Larger short-channel effects.
